# Attachment style and addictions (alcohol, cigarette, waterpipe and internet) among Lebanese adolescents: a national study

**DOI:** 10.1186/s40359-020-00404-6

**Published:** 2020-04-16

**Authors:** Laurette Nakhoul, Sahar Obeid, Hala Sacre, Chadia Haddad, Michel Soufia, Rabih Hallit, Marwan Akel, Pascale Salameh, Souheil Hallit

**Affiliations:** 1 Faculty of Medicine and Medical Sciences, Holy Spirit University of Kaslik (USEK), Jounieh, Lebanon; 2 Research and Psychology Departments, Psychiatric Hospital of the Cross, P.O. Box 60096, Jall-Eddib, Lebanon; 3 Faculty of Arts and Sciences, Holy Spirit University of Kaslik (USEK), Jounieh, Lebanon; 4 INSPECT-LB: Institut National de Santé Publique, Epidemiologie Clinique et Toxicologie, Beirut, Lebanon; 5 Drug Information Center, Order of Pharmacists of Lebanon, Beirut, Lebanon; 6 Univ. Limoges, UMR 1094, Neuroépidémiologie Tropicale, Institut d’Epidémiologie et de Neurologie Tropicale, GEIST, 87000 Limoges, France; 7 Faculty of Medicine, Lebanese University, Hadat, Lebanon; 8 School of Pharmacy, Lebanese International University, Beirut, Lebanon; 9 Faculty of Pharmacy, Lebanese University, Hadat, Lebanon

**Keywords:** Adolescents, Attachment styles, Addiction, Internet, Cigarette, Waterpipe, Alcohol

## Abstract

**Background:**

The prevalence in the Lebanese general population of cigarette and waterpipe smoking, alcohol drinking and internet use seems to be increasing lately. So far, no study was done relating the above to attachment styles in Lebanese adolescents. Consequently, the objective of our study was to assess the relationship between attachment styles (secure, preoccupied, fearful, and dismissing) and addictions (cigarettes, water pipes, alcohol, and internet) among this population.

**Methods:**

It is a cross-sectional study that took place between January and May 2019. Two thousand questionnaires were distributed out of which 1810 (90.5%) were completed and collected back. A proportionate random sample of schools from all Lebanese Mohafazat was used as recruitment method.

**Results:**

A secure attachment style was significantly associated with lower addiction to alcohol, cigarette, and waterpipe, whereas insecure attachment styles (preoccupied, dismissing and fearful) were significantly associated with higher addiction to cigarette, waterpipe, alcohol, and internet.

**Conclusion:**

Lebanese adolescents with insecure attachment had higher rates of addiction to cigarette, waterpipe, alcohol, and internet. They should be closely monitored in order to reduce the risk of future substance use disorder and/or behavioral addiction development.

## Background

Originally proposed [1], then developed by John Bowlby [2] and Mary Ainsworth [3], the attachment theory explains the effect of interpersonal relationships on normal and abnormal psychological functioning. According to Bowlby, the quality of close relationships between people, from early childhood, interferes in the elaboration of mental representations of oneself and others. These representations constitute the roots for social experiences and environmental understandings. This being said those representations might be the source of vulnerability in the case of personal history of insecure relationships, or on the contrary source of resilience in the case of secured ones [4]. Four types of attachment styles were identified: secure, anxious or preoccupied, dismissive, and fearful. Securely attached people are optimistic about themselves and others [5]; their caregivers were emotionally stable and provided them with quality time during childhood, thereby regulating their positive and negative emotions [6]. Preoccupied people are optimistic about others but pessimistic about themselves, hence worried about relationships [5]. Dismissive people are optimistic about themselves but pessimistic about others, therefore, do not value relations [5]. Fearful people are pessimistic about themselves and others; they think they are unlovable and untrustworthy, and others will reject them [5].

According to Attachment Theory, humans are biologically prone to create strong bonds with other humans from whom they receive emotional support and protection [1]. The type of attachment that will an infant develop towards its primary caregiver will have future implications on his social and emotional functioning at an older age. These ties will play vital roles throughout the life of individuals [7]. As children grow, they develop new attachment relationships with friends and romantic partners [8], and most adults report using both parents and their romantic partners as attachment figures [9]. In other words, the formation of attachment is a process of development that persists well beyond infancy and childhood [10]. During adolescence, representations of attachment relationships can be continually changed as individuals develop new intimate relationships [11]. This process of change is not only the product of the adolescent’s autonomy and increased physical abilities, but also of cognitive development that marks the transition to formal operations, which greatly increases the individual’s ability to think about his motivations and interpersonal relationships [12]. Alongside the increase in the adolescent’s abstract thinking capacity, there are many environmental issues, including the transition to university, the development of more intimate relationships, concerns about self-image and puberty, possible family problems, and the development of sexuality [13]. This association of increased abilities and increased environmental constraints during adolescence seems to create ideal conditions for the development of a wider range of intimate relationships, and vulnerability to the development of dysfunctional behavior, such as addiction [1, 14] that is defined by the American Society of Addiction Medicine (ASAM) as “*a primary chronic disease of brain reward, motivation, memory and related circuitry*” [15]. Many believe that addictions are a coping mechanism and that the attachment style can play a key role in the development of addictions [16], such as those related to smoking, alcohol, and internet.

Previous findings suggest that smokers were more likely to be of the anxious type [17]. Indeed, young adults who tend to smoke have more conflicts within their families and are, therefore, influenced by external objects, such as cigarettes to overcome their psychological needs [18], but to our knowledge, no studies have shown an association between waterpipe addiction in adolescence and attachment styles.

As for alcohol use disorder, previous studies revealed that subjects addicted to alcohol and other psychoactive substances are very likely to have insecure and avoidance attachment styles [19, 20], in addition to higher anxiety-trait, alexithymia, and schizoid traits [19, 21].

Moreover, a correlation was found between insecure attachment style and internet addiction [22]. In fact, the secure type was shown to be a protective factor against internet addiction since people with this attachment style have a high self-esteem in face-to-face relationships [23]. In contrast, the anxious type was associated with higher social networking addiction because people of this type make huge efforts to be accepted by others. They also fear face-to-face interactions and have, through social networking, the advantage of choosing the time for interacting with other people and the way they present themselves to others [23, 24]. The avoidant type is also correlated with higher social networking addiction since these people satisfy their social needs through online platforms while keeping a safe distance [23].

The incidence of cigarette smoking is increasing globally, with a higher incidence in developing countries [25], where Lebanon ranks first in terms of smoking prevalence in the Middle-East and among Arab women [25]. Its prevalence is also rising among adolescents (3.9% frequent smokers in 2012), especially in the presence of a family member or friend who smokes [26]. Similarly, the prevalence of waterpipe use is high in Lebanon, with up to 35% of the 13 to 15 years old category having already used it [27]. Actually, waterpipes are more appealing to adolescents due to the different flavors and look, and due to the false idea of waterpipe smoking being a non-harmful method [28], its use in adolescent population is popular [29, 30]. Its popularity among adolescents might be also due to its social acceptance [31]. This also leads to an increase in its prevalence (19% of Lebanese adolescents were frequent waterpipe users in 2012) [26]. Moreover, the lack in the application of the laws protecting minors from drinking alcohol makes it easier for them to buy it with no respect to age restrictions. This, combined with alcohol cheap prices in Lebanon play a role in the increase of alcohol consumption in this category [32]. Alcohol use among adolescence is due to curiosity, power feeling, media influence and to cope with stress [33, 34]. This might explain the increased alcohol addiction in adolescents (an increase in Lebanese adolescent lifetime drunkenness between 2005 and 2011 by 48%) [35]. As for Internet addiction, studies revealed that the global prevalence is 1.6 to 18% [36] and varies with sex, age, and ethnicity [37]. In Lebanon, it is around 40–42% among adolescents [38, 39]. High incidence is explained by the fact that social media is used to enhance friendly relationships [40] whereas gaming activities, interactive platforms, hook adolescents to the internet leading to an increased risk of addiction by increasing internet usage time [41].

In addition to all these factors, Lebanese adolescents [42] and adults [43] have to face many other stressors leading them to high levels of exclusion and destabilization, such as the Syrian crisis and the resulting immigration, internal instability, and the persistent economic inequalities. Moreover, Lebanese adolescents have a low level of participation and civic engagement and feel powerless in the political system due to lack of representation, low budget allocation, and weak implementation of policies and programs for youth (Unicef Lebanon). All these factors can increase the risk of addiction among adolescents, with previous studies showing that adolescents with anxious attachment may have alcohol problems [44]. In such settings, and since the prevalence in the Lebanese general population of cigarette and waterpipe smoking, alcohol drinking and internet use seems to be increasing lately [45, 46], and in the absence of studies on their association with attachment styles, it decision to conduct such a research among Lebanese adolescents was taken. Consequently, the objective of our study was to assess the relationship between attachment styles (secure, preoccupied, fearful, and dismissing) and addictions (cigarettes, water pipes, alcohol, and internet) among Lebanese adolescents.

## Methods

### Participants

This cross-sectional study was conducted between January and May 2019. A proportionate random sample of schools from all Lebanese Mohafazat (Beirut, Mount Lebanon, North, South and Bekaa) was used as recruitment method. The list of schools was obtained from the Ministry of Education and Higher Education in Lebanon. From each mohafaza, a proportionate number to the total number of schools was selected; in case of school refusal to participate, replacement was not done. Out of the of the 18 private schools contacted, 2 refused to participate, and the 16 that accepted were located as follows: 4 in Beirut, 2 in South Lebanon, 6 in the Mount Lebanon, 2 in North Lebanon, and 2 in Bekaa. All students, aged between 14 and 17 years old, from each school were eligible to participate. Students who refused to fill the questionnaire were excluded. The methodology used has been previously described [39, 44, 47–72].

### Minimal sample size

Since there are no similar studies in Lebanon, we postulated that an insecure attachment style would increase waterpipe dependence moderately (effect size r = 0.3). The G-power software estimated a minimal sample of 134 participants (power of 95%). The total sample at the end of the data collection consisted of 1810 (90.5%) questionnaires collected back out of 2000 distributed.

### Questionnaire

A study-independent personnel was responsible for the distribution of the questionnaire. It was in Arabic, the native language of Lebanon, and required approximately 30 min to complete. Students were asked to fill out the questionnaire during classes to avoid parental influence in answering questions. The anonymity of the participants was guaranteed during the data collection process.

The sociodemographic details of the participants were addressed in the first part of the questionnaire (i.e. age, gender, smoking status). Participants self-reported their heights and weights based on which the Body Mass Index (BMI) (kg/m^2^) was calculated. The household crowding index was calculated by dividing the number of persons living in the house by the number of rooms in the house, excluding the bathroom and the kitchen [73].

The second part of the questionnaire included the following scales:

#### Relationship questionnaire (RQ)

This questionnaire consists of four short paragraphs, each describing one of the four adult attachment styles. Style A relates to the secure attachment, Style B to the preoccupied attachment, Style C to the fearful attachment, and Style D to the dismissing attachment [74]. Each paragraph is rated on a 7-points Likert scale (1 = strongly disagree to 7 = strongly agree) (α_Cronbach_ in this study = 0.970).

#### Internet addiction test (IAT)

This 20-item tool is rated on a 6-point Likert scale from 0 = does not apply/never to 5 = always applies [75]. The total score varied between 20 and 100, with higher scores defining higher internet addiction (α_Cronbach_ in this study = 0.925). This scale has previously been validated among Lebanese adolescents [76].

#### The alcohol use disorders identification test (AUDIT)

This self-reported screening tool consists of 10 items and assesses alcohol use, drinking patterns, and alcohol-related issues [77]. The AUDIT scale was shown to have an acceptable sensitivity for the identification of alcohol problems in adolescents aged between 14 and 18 years old [78]. A score equal to or greater than 8 indicates the presence of an alcohol use disorder (α_Cronbach_ in this study = 0.960).

#### Lebanon Waterpipe dependence Scale-11 (LWDS-11)

This test is used to assess waterpipe dependence [79]. It includes 11 items measured on a 4-point Likert scale ranging from 0 to 3; higher scores reflect higher waterpipe dependence (α_Cronbach_ in this study = 0.888).

#### Fagerström test for nicotine dependence (FTND)

This 6-item tool is used to assess the intensity of physical addiction to nicotine related to cigarette smoking. The higher the total score, the more intense the patient’s physical dependence on nicotine [80]. This scale can be used to asses cigarette addiction in adolescents [81] (α_Cronbach_ in this study = 0.825).

### Translation procedure

The translation from English to Arabic was carried out by a single bilingual translator. A backward translation was then performed by a native English-speaking translator, fluent in Arabic and unfamiliar with the concepts of the scales. Discrepancies were resolved by consensus between translators and researchers.

### Statistical analysis

Data analysis was performed using SPSS software version 23. Cronbach’s alpha values were recorded for reliability analysis for all the scales. Missing data was not replaced since it formed less than 10% of the total data. Attachment styles measures were then dichotomized according to the mean. For bivariate analysis, the Student’s test was used to compare means between two groups, and correlation coefficients were used to assess the association between continuous variables. In all cases, a *p*-value lower than 0.05 was considered significant. A multivariate analysis of covariance (MANCOVA) was carried out to compare multiple measures (each addiction scale was taken as a dependent variable) between the dichotomized attachment styles categories, taking into account potential confounding variables: age, gender, house crowding index and BMI. A *p* < 0.05 was considered significant.

## Results

The sociodemographic characteristics of the participants are summarized in Table 1. The mean age was 15.42 ± 1.14 years, with 53.3% females. The means and standard deviations for the scales were as follows: AUDIT (6.46 ± 8.44), IAT (39.42 ± 18.08), FTND (1.53 ± 2.83) and LWDS (4.73 ± 8.68). Furthermore, the results showed that 43.45% of the participants had a secure attachment style [95% CI 0.408–0.461], 44.18% a preoccupied style [95% CI 0.442–0.415], 49.01% a fearful style [95% CI 0.464–0.517] and 45.50% a dismissing style [95% CI 0.429–0.481].

**Table 1:** Sociodemographic characteristics of the sample population

	**Frequency (%)**
**Gender**	
Male	844 (46.7%)
Female	963 (53.3%)
**Smoking status**	
Yes	468 (25.9%)
No	1342 (74.1%)
	**Mean ± SD**
**Age (years)**	15.42 ± 1.14
**Body Mass Index (kg/m2)**	21.95 ± 4.21
**Household crowding index**	1.01 ± 0.64

### Bivariate analysis

No significant difference was found between genders in terms of alcohol use disorder, cigarette, and waterpipe dependence (Table 2). Higher internet addiction, cigarette and waterpipe dependence, fearful style, and dismissing style were significantly associated with higher alcohol use disorders, whereas higher secure and preoccupied styles were significantly associated with lower alcohol use disorders. Higher cigarette and waterpipe dependence, higher age, preoccupied and fearful styles were significantly associated with higher internet addiction. Higher waterpipe dependence, preoccupied, fearful, and dismissing styles were significantly associated with higher cigarette dependence, whereas higher age, higher house crowding index, and higher secure style were significantly associated with lower cigarette dependence. Higher body mass index, preoccupied, fearful, and dismissing styles were significantly associated with higher waterpipe dependence, whereas higher age, house crowding index, and secure style were significantly associated with lower waterpipe dependence (Table 3).

**Table 2:** Bivariate analysis of categorical variables associated with the addictions scores

Variable	AUDIT	IAT	FTND	LWDS
**Gender**				
Male	6.04 ± 8.43	39.07 ± 18.62	1.50 ± 2.74	5.00 ± 8.87
Female	6.82 ± 8.43	39.73 ± 17.60	1.56 ± 2.91	4.50 ± 8.51
*p*-value	0.056	0.444	0.648	0.225

*AUDIT* Alcohol Use Disorder scale, *IAT* Internet Addiction Test, *FTND* Cigarette dependence scale, *LWDS* Waterpipe Dependence scale.

Scales range: AUDIT (0–40), IAT (0–100), FTND (0–10), LWDS (0–33).

**Table 3:** Bivariate analysis of continuous variables associated with the addictions scores

Variable	AUDIT	IAT	FTND	LWDS
AUDIT	1			
IAT	0.325^c^	1		
FTND	0.576^c^	0.108^c^	1	
LWDS	0.523^c^	0.05^a^	0.782^c^	1
Age	0.009	0.052^a^	−0.147^c^	−0.152^c^
House crowding index	−0.03	− 0.012	−0.089^c^	− 0.081^b^
Body Mass Index	0.013	0.019	0.035	0.147^c^
Relationship style A- secure	−0.222^c^	0.013	−0.210^c^	−0.084^b^
Relationship style B- preoccupied	−0.143^c^	0.180^c^	0.058^a^	0.016
Relationship style C- fearful	0.170^c^	0.155^c^	0.226^c^	0.151^c^
Relationship style D- dismissing	0.194^c^	0.041	0.291^c^	0.251^c^

*AUDIT* Alcohol Use Disorder scale, *IAT* Internet Addiction Test, *FTND* Cigarette dependence scale, *LWDS* Waterpipe Dependence scale.

^a^*p* < 0.001

^b^*p* < 0.01

^c^*p* < 0.05

### Addiction scores means according to attachment styles

**Fig. 1:**
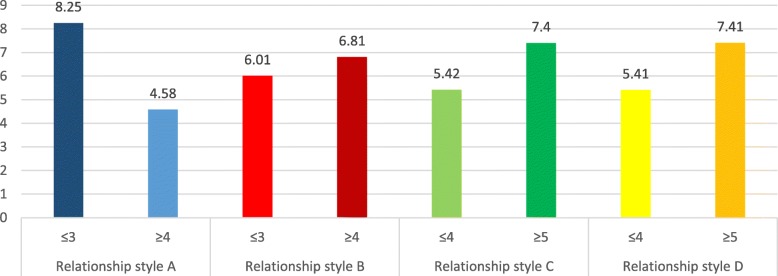
Adjusted means for alcohol use disorders scores per attachment style. Style A: secure attachment; Style B: preoccupied attachment; Style C: fearful attachment; Style D: dismissing attachment

**Fig. 2:**
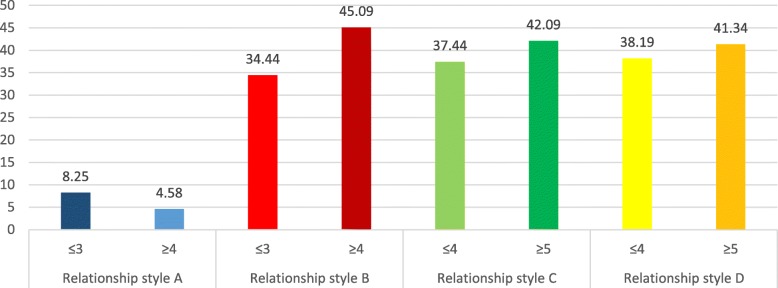
Adjusted means for internet addiction scores per attachment style. Style A: secure attachment; Style B: preoccupied attachment; Style C: fearful attachment; Style D: dismissing attachment

**Fig. 3:**
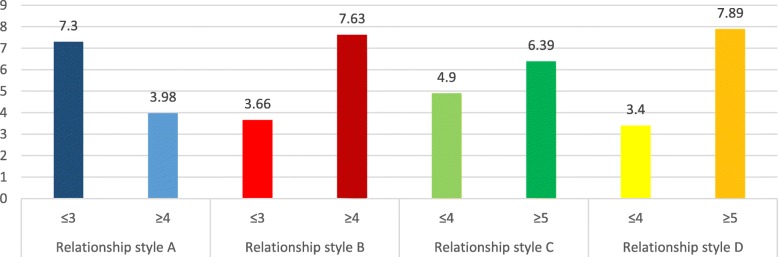
Adjusted means for the waterpipe dependence scores per attachment style. Style A: secure attachment; Style B: preoccupied attachment; Style C: fearful attachment; Style D: dismissing attachment

**Fig. 4:**
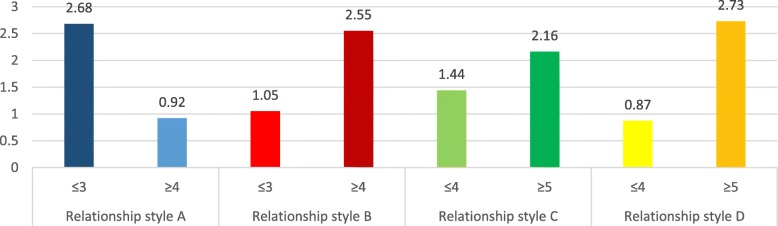
Adjusted means for the cigarette dependence scores per attachment style. Style A: secure attachment; Style B: preoccupied attachment; Style C: fearful attachment; Style D: dismissing attachment

The adjusted means for the addiction scores according to each attachment style are summarized in Figs. 1, 2, 3 and 4. In all types of addiction, a secure attachment style is associated with lower addiction, while unsecured attachment styles were significantly associated with higher addiction.

### Multivariate analysis

The MANCOVA analysis was performed taking the scales as the dependent variable and the attachment styles as independent variables, after adjusting for the covariates (age, gender, house crowding index and BMI).

A secure relationship style was significantly associated with lower AUDIT scores (B = -3.35), lower cigarette (B = -1.57) and waterpipe (B = -2.73) dependence. A fearful relationship style was significantly associated with higher AUDIT scores (B = 1.83), internet addiction (B = 4.46) and cigarette dependence (B = 0.58). A dismissing relationship style was significantly associated with higher AUDIT scores (B = 1.79), internet addiction (B = 2.64), cigarette (B = 1.77) and waterpipe (B = 4.23) dependence. Finally, a preoccupied relationship style was significantly associated with higher internet addiction (B = 8.84), cigarette (B = 1.43) and waterpipe (B = 3.95) dependence (Table 4).

**Table 4:** Multivariate analysis of covariance (MANCOVA)

	Beta	***p***-value	95% Confidence Interval	
			Lower Bound	Upper Bound
**AUDIT total score**				
Gender (males vs females^a^)	−1.33	0.002	−2.18	−0.49
Secure relationship style (yes vs no^a^)	−3.35	< 0.001	−4.37	−2.33
Fearful relationship style (yes vs no^a^)	1.83	0.001	0.72	2.93
Dismissing relationship style (yes vs no^a^)	1.79	< 0.001	0.80	2.78
**IAT total score**				
Preoccupied relationship style (yes vs no^a^)	8.84	< 0.001	6.13	11.55
Dismissing relationship style (yes vs no^a^)	2.64	0.031	0.24	5.04
Fearful relationship style (yes vs no^a^)	4.46	0.001	1.78	7.13
**FTND**				
Gender (males vs females^a^)	−0.35	0.025	−0.66	− 0.05
Secure relationship style (yes vs no^a^)	−1.57	< 0.001	− 1.94	− 1.20
Preoccupied relationship style (yes vs no^a^)	1.43	< 0.001	1.03	1.84
Fearful relationship style (yes vs no^a^)	0.58	0.005	0.18	0.99
Dismissing relationship style (yes vs no^a^)	1.77	< 0.001	1.41	2.13
Age	−0.39	< 0.001	−0.52	− 0.26
Household crowding index	−0.30	0.007	−0.51	− 0.08
**LWDS-11**				
Secure relationship style (yes vs no^a^)	−2.73	< 0.001	−3.86	−1.60
Preoccupied relationship style (yes vs no^a^)	3.95	< 0.001	2.71	5.19
Dismissing relationship style (yes vs no^a^)	4.23	< 0.001	3.14	5.33
Age	−1.21	< 0.001	−1.61	−0.82
Household crowding index	−0.72	0.032	−1.37	−0.06

^a^Reference group

*AUDIT* Alcohol Use Disorder scale, *IAT* Internet Addiction Test, *FTND* Cigarette dependence scale, *LWDS* Lebanese waterpipe Dependence scale.

## Discussion

This is the first study of its kind in Lebanon to address the association between the attachment styles and addictions in adolescents. A secure attachment style was significantly associated with lower addiction to alcohol, cigarette, and waterpipe, whereas insecure attachment styles (preoccupied, dismissing and fearful) were significantly associated with higher addiction to cigarette, waterpipe, alcohol, and internet.

A secure attachment style, increased age, and higher house crowding index were associated with lower cigarette smoking addiction in adolescents, as opposed to insecure attachment styles (preoccupied, dismissing, and fearful) and female gender, in line with previous research [18, 82]. Indeed, people with insecure attachment styles have low self-esteem and more anxiety, which increase substance abuse, such as smoking, especially in cases of emotional distress [83]. Further, people with insecure attachment (high avoidance/anxiety) have a lower capability to self-regulate during stressful times/situations and consequently look for external methods to relieve their stress, such as tobacco use; hence, tobacco use can be considered as a coping mechanism for stress in those persons [84]. The negative association between increased house crowding index and smoking can be hypothesized by the fact that a crowded house does not give to adolescents freedom or the time to smoke alone, especially if their roommate is annoyed by passive smoking.

Waterpipe addiction was positively associated with all insecure attachment styles and negatively associated with increased age. This could be due to the same reasons related to cigarette smoking. According to studies, a 45-min waterpipe smoking is equivalent to approximately 40 cigarettes [85]. These high nicotine levels in waterpipe are believed to relieve anxiety and mitigate psychological problems [86]. Another factor that has significantly contributed to the increased use of the waterpipe is the perception of its “positive” attributes, such as socializing, relaxing, and the good taste/smell of the smoke; these attributes seem to encourage and maintain waterpipe use, thereby reducing anxiety [87]. However, as adolescents grow older, they learn more about the side effects of waterpipe and cigarette smoking, which can explain the negative association between aging and both smoking addictions [88].

Our results also show that the secure attachment style was negatively associated with alcohol addiction, contrary to insecure attachment styles, consistent with previous findings on adults [44]. This is probably due to the fact that people with insecure attachment styles have problems with anxiety and relationship stability, which increases the risk of any substance abuse, including alcohol [19]. Alcohol is a substrate of the GABAergic receptors that decreases anxiety and controls emotions; this means that people with an insecure attachment style will feel more relieved by drinking alcohol, which can lead to addiction over time [19]. Female gender was also associated with alcohol addiction. In fact, alcohol is absorbed faster and metabolized slower in women [89], leading to higher blood alcohol concentrations in women for a longer time with the same amount of ingested alcohol, thereby increasing the risk of addiction.

As for internet addiction, it was positively related with insecure attachment styles (preoccupied and dismissing) in adolescents, consistent with previous studies [16]. People with an insecure style have problems with self and/or others’ image, thus hindering direct relationships [5]. Consequently, they prefer indirect relationships, using social media, to face-to-face relationships (e.g. Facebook); this will increase the time spent on social media, resulting in an increased risk of internet addiction [23, 24]. On another hand, people with a secure attachment style have high self-esteem and do not have any difficulty sharing their feelings with others, making them able to manage time spent on social media without becoming addict [7].

### Clinical implications

The relationship between insecure attachment styles and addictions require specific treatment considerations. Thus, it appears reasonable to take therapeutic actions to support persons reporting real-life shortfalls; this can be achieved either by a good patient-communication relationship, which can be considered as a “substitute attachment figure” or by a group therapy that can also provide corrective relationship experiences. In addition, prevention programs are warranted, and healthcare providers could explore tools to build a more secure attachment in children to possibly decrease the likelihood of future addictions (tobacco, waterpipe, alcohol and internet addiction) initiation.

### Limitations

This study has few limitations. Its cross-sectional nature does not allow inferring causality due to temporality issues. The Relationship Questionnaire is a very reductive form of measurement for adult attachment and close relationships. It was not validated among adolescents in Lebanon, which may lead to a non-differential information bias, underestimating the relationship between attachment style and addictive behaviors. All scales used, except the IAT, have not been validated among Lebanese adolescents, which might have led to a non-differential information bias; this might explain the negative correlation between age with smoking (cigarettes and waterpipe). This finding, in itself, could be pointing to the issue of addiction needing to be measured differently during adolescence due to the tendency for adolescents to experiment this kind of addiction during this period of development. Information bias might be present because of trouble understanding a question. The study did not include adolescents not attending schools, which hinders extrapolation to out-of-school adolescents where addiction problems might be expected to be more common. Finally, a selection bias might be present because of the selection process of schools since public schools were not included. However, we believe that our results are generalizable to the whole adolescents’ population in Lebanon.

## Conclusion

In conclusion, Lebanese adolescents with insecure attachment had higher rates of addiction to cigarette, waterpipe, alcohol, and internet. They should be continuously followed-up to avoid developing any addiction that could potentially harm them. Future studies that include out-of-school adolescents are warranted to confirm our findings.

## Data Availability

Data can be made available under reasonable request form the corresponding author.

## Abbreviations

ASAM: American Society of Addiction Medicine
RQ: Relationship questionnaire
IAT: Internet Addiction Test
AUDIT: Alcohol Use Disorders Identification Test
LWDS: Lebanon waterpipe dependence scale
FTND: Fagerström test for nicotine dependence
